# Why has the Universal Coverage Scheme in Thailand achieved a pro-poor public subsidy for health care?

**DOI:** 10.1186/1471-2458-12-S1-S6

**Published:** 2012-06-22

**Authors:** Supon Limwattananon, Viroj Tangcharoensathien, Kanjana Tisayaticom, Tawekiat Boonyapaisarncharoen, Phusit Prakongsai

**Affiliations:** 1 Khon Kaen University, Thailand; 2 International Health Policy Program, Ministry of Public Health, Thailand; 3 Office of Health Inspector, Ministry of Public Health, Thailand

## Abstract

**Background:**

Thailand has achieved universal health coverage since 2002 through the implementation of the Universal Coverage Scheme (UCS) for 47 million of the population who were neither private sector employees nor government employees. A well performing UCS should achieve health equity goals in terms of health service use and distribution of government subsidy on health. With these goals in mind, this paper assesses the magnitude and trend of government health budget benefiting the poor as compared to the rich UCS members.

**Method:**

Benefit incidence analysis was conducted using the nationally representative household surveys, Health and Welfare Surveys, between 2003 and 2009. UCS members are grouped into five different socio-economic status using asset indexes and wealth quintiles.

**Findings:**

The total government subsidy, net of direct household payment, for combined outpatient (OP) and inpatient (IP) services to public hospitals and health facilities provided to UCS members, had increased from 30 billion Baht (US$ 1 billion) in 2003 to 40-46 billion Baht in 2004-2009. In 2003 for 23% and 12% of the UCS members who belonged to the poorest and richest quintiles of the whole-country populations respectively, the share of public subsidies for OP service was 28% and 7% for the poorest and the richest quintiles, whereby for IP services the share was 27% and 6% for the poorest and richest quintiles respectively. This reflects a pro-poor outcome of public subsidies to healthcare. The OP and IP public subsidies remained consistently pro-poor in subsequent years.

The pro-poor benefit incidence is determined by higher utilization by the poorest than the richest quintiles, especially at health centres and district hospitals. Thus the probability and the amount of household direct health payment for public facilities by the poorest UCS members were less than their richest counterparts.

**Conclusions:**

Higher utilization and better financial risk protection benefiting the poor UCS members are the results of extensive geographical coverage of health service infrastructure especially at district level, adequate finance and functioning primary healthcare, comprehensive benefit package and zero copayment at points of services.

## Background

Thailand has achieved universal health coverage since 2002 through the implementation of the Universal Coverage Scheme (UCS) for 47 million (75%) of the population who were neither private sector employees covered by the Social Health Insurance Scheme, nor government employees and dependants covered by the Civil Servant Medical Benefit Scheme [1].

**Figure 1:**
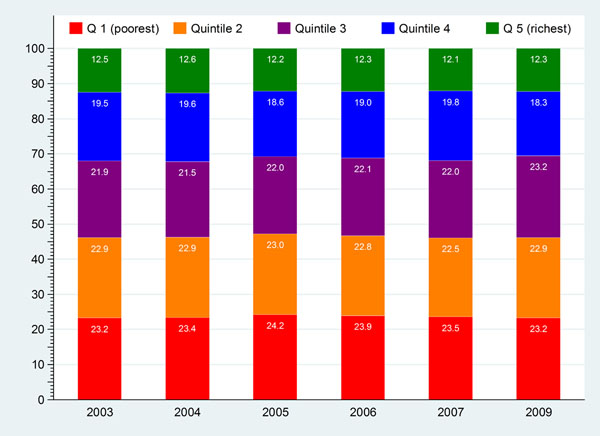
National quintiles of household asset index of UCS beneficiaries as % of all members, 2003-2009

Grouping 47 million UCS members of the whole-country’s 65 million population by their households’ asset index quintiles (20 percent equally), the poorest and poor quintiles accounted for almost half (46-47%) of all UCS members (Figure 1) while less than 15% of the UCS beneficiaries belonged to the richest quintile between 2003 and 2009. The UCS covers most of the poor Thai population who reside in the rural areas. Policy concerns if the poor UCS members benefit from the Scheme.

Increased utilization of services among UCS members was observed; the total outpatient (OP) visits to district and provincial hospitals increased from 111.9 million visits in 2003 to 140.7 million in 2009 [2]. Total inpatient (IP) admissions to the Ministry of Public Health (MOPH) hospitals also increased from 4.30 million in 2003 to 5.21 million in 2009 [3].

A rigorous analysis of the impact of the UCS over the period of 1996 to 2005 [covering the period prior to and after achieving UC in 2002] revealed a reduction in probability of not seeking care when ill and not using health care from the informal sector providers for outpatient services by 1.3 and 2.3%, respectively [4]. The probability of having a visit to district hospitals increased by 2.3% and to provincial hospitals reduced by 4.1%. This is a result of the policy to promote primary care as the first contact when ill. For those who were hospitalized, the probability of using district hospitals increased by 3.5%, while the use at provincial hospitals reduced by 6.4%.

Prior studies on the whole-country and all-scheme population revealed the OP and IP utilization concentrated more among the poor than the rich and the government health subsidy was pro-poor [5,6]. A well performing UCS should be able to achieve health equity goal. With this goal in mind, this study assessed the magnitude and trend of health budget distribution and whether it benefited the rich or the poor UCS members at national and sub-national levels, and discussed factors contributing to the pro-poor subsidies.

## Methods

Secondary data analyses used a benefit incidence analysis (BIA) approach to assess the distribution of government subsidies on health to different socio-economic groups who were UCS members. BIA is defined as “a method of computing the distribution of public expenditure across different demographic groups, such as women and men”. The procedure involves allocating per unit public subsidies, for example, expenditure per student for the education sector, according to individual utilization rates of public services [7].

### Data sources

A series of Health and Welfare Surveys (HWS) was used from 2003, one year following the nationwide implementation of UCS to the most recent year in 2009, noting that HWS was conducted every year for five years between 2003 and 2007 and thereafter every two years in 2008 and beyond. The HWS is a structured household interview survey on illnesses and health service utilization of approximately 70 thousand individuals from nationally representative households. The Survey used to be conducted every five years before the UCS period by the National Statistical Office (NSO). Unfortunately prior to the advent of UCS in 2002, a number of key variables in the HWS were missing, notably quantification of health service utilization, health payments and economic status of the interviewees and households.

A one-month recalling period for non-hospitalized and one year for hospitalized illnesses were applied. Choices for the ambulatory care for the last illness episode include the whole range of providers not covered by UCS such as self medication, herbal medicine and traditional healers as well as health facilities covered by UC such as health centres, district hospitals, provincial hospitals, university hospitals, other government hospitals, private clinics and hospitals. Choices of the hospitalization include both public and private hospitals excluding health centres and private clinics which do not provide inpatient services. Out-of-pocket payments by household members for each OP visit and IP admission were asked in monetary terms.

### Equity stratifier

To determine the economic gradient of UCS members, this analysis used a presence (or an absence) of selected assets in households. Wealth better reflects living standards than incomes since wealth reflects both inventory and flows. A person living in the household constructed with permanent materials and having toilets, motor vehicles and electrical equipments such as televisions, telephones, refrigerators and computers was considered richer than those who do not own these assets. Through a principal component analysis [8] of all household samples, an asset index was created for each household. All households in the country were then ranked by asset indices of their households then divided into five subgroups equally into five wealth quintiles, using the individual sampling weights. Only the UCS beneficiaries with respect to the national wealth quintiles of their households were selected for this analysis.

### Health service utilization

The health care utilization in the analysis was limited to services covered by UCS, for which self medication at pharmacies or drug stores, traditional medicines, and traditional healers were not covered.

The number of OP visits was obtained from the utilization of services among household members reporting non-hospitalized illnesses with up to 8 possible episodes in the last month prior to the interview. The annualized (12-month) OP visits per facility type were then calculated. The number of hospital admissions per year for each health facility was directly estimated from the number of reported admissions in the last year.

### Government health subsidy

Since public hospitals and facilities are the typical providers of UCS members where budget was allocated based on capitation for OP services and case-based payment under global budget for IP services, these public providers were included in the assessment of government health subsidies.

The UCS-specific unit costs per OP visit and per IP admission were obtained from National Health Security Office (NHSO). It is noted that university hospitals had the highest cost 1,600-1,900 Baht per OP visit and 18,000-27,000 Baht per admission compared with the lower level facilities.

The unit cost that NHSO paid for an OP and IP service, netted out as direct payment for that patient was defined as the unit subsidy. If the amount of payment was greater than the unit cost, the subsidy became zero. The amount of government health subsidy was calculated by multiplying the unit subsidy with the number of OP visits or IP admissions at different types of health facility by each individual UCS member. The facility-specific subsidies for OP and IP services were summed up and compared across different wealth quintiles.

## Findings

### Profiles of government health subsidies

#### National aggregate

The amount of government health subsidy, net of direct household payment, to UCS beneficiaries was 30.15 billion Baht in 2003 at 2009 price (Table 1) or US$ 1 billion (exchange rate, 30 Baht per dollar); it increased to 46.5 billion Baht in 2009, a 36.2% real term increase. It is noted that subsidy for the OP was lower than IP services.

**Table 1:** Amount of government health subsidy by types of health service and facility, 2009 constant price

	2003	2004	2005	2006	2007	2009
1. Total subsidy, million Baht	34,147	42,300	46,838	44,812	47,505	46,502

**1.1 Total OP subsidy**	**15,092**	**16,355**	**16,643**	**14,341**	**14,862**	**20,489**

○ Health center	2,363	2,634	2,955	1,656	1,931	2,339

○ District hospital	7,269	7,439	7,847	7,227	6,422	8,523

○ Provincial hospital	3,395	3,994	4,432	4,430	4,642	7,674

○ University hospital	1,001	1,516	818	626	1,037	736

○ Other govt. hospital	1,063	773	589	401	829	1,217

**1.2 Total IP subsidy**	**19,055**	**25,943**	**30,195**	**30,471**	**32,643**	**26,013**

○ District hospital	7,840	9,117	10,081	10,432	9,969	7,322

○ Provincial hospital	8,115	13,054	15,049	16,364	19,521	16,749

○ University hospital	759	2,496	3,082	2,157	1,747	699

○ Other govt. hospital	2,340	1,276	1,984	1,518	1,405	1,242

						

2. Total subsidy, Baht per capita	708	884	1,016	945	998	947

**2.1 OP subsidy per capita**	**313**	**342**	**361**	**302**	**312**	**417**

○ Health center	49	55	64	35	41	48

○ District hospital	150	155	171	153	135	174

○ Provincial hospital	70	83	96	94	97	156

○ University hospital	21	32	18	13	21	15

○ Other govt. hospital	22	16	13	8	17	25

**2.2 IP subsidy per capita**	**395**	**542**	**655**	**642**	**686**	**530**

○ District hospital	162	191	219	220	210	149

○ Provincial hospital	169	273	326	344	410	341

○ University hospital	16	52	67	45	37	14

○ Other govt. hospital	49	27	43	32	30	25

Subsidies by level of care, the majority of the UCS budget subsidized OP services at district hospitals, followed by provincial hospitals, especially in recent years. The subsidy to health centers did not show a noticeable increase due to a lower unit cost and a slower increasing trend of utilization. Provincial hospitals had a major share of the IP subsidy and the trend is increasing, due to the higher unit cost than district hospitals and more provision of tertiary care services with higher cost.

On average, the government subsidy for all-facility OP visit and IP admission was 313 and 395 Baht per UCS beneficiary respectively in 2003; this had increased to 417 and 530 Baht respectively in 2009.

**Figure 2:**
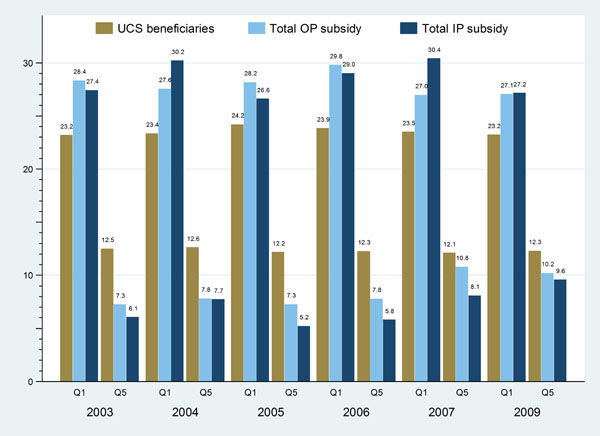
Distribution of OP and IP government subsidies by wealth quintile as compared with the UCS beneficiary distribution, 2003-2009

Subsidies by wealth quintiles, subsidy for OP service used by the poorest quintile UCS beneficiaries was 27-30% compared to their respective share of members, 23-24% of total UC population; while the richest quintiles reaped the benefit of only 7-11% of the total subsidies, less than the population share, 12-13% of total UC members (Figure 2).

The pro-poor pattern also emerged for IP subsidies; 27-30% of government health budget net of household payment went to the poorest quintile, which is higher than the size of the poorest quintile; while only 6-10% went to the richest quintile in these years, lower than the size of the richest quintile.

#### Subsidy by type of health facility

**Figure 3:**
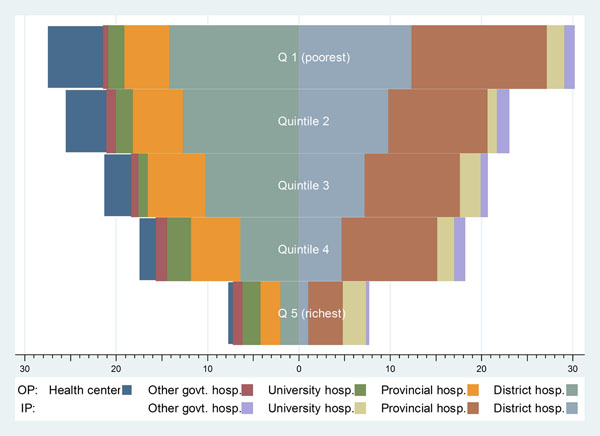
Distribution of OP and IP subsidies by facility type for each wealth quintile, 2004

Figure 3 shows distribution of OP and IP subsidies across wealth quintiles of the UCS beneficiaries with respect to health facility types. A similar pattern emerged in 2003-2009; data from 2004 were selected for illustration. Clearly, the poorest quintiles benefit most of the OP and IP subsidies while the richest quintiles benefit the least.

Even though health centers had a relatively a larger share of the OP visits, especially for the lower quintiles, their subsidy share was noticeably smaller than that of district and provincial hospitals due to their much lower OP unit costs. A similar explanation was applied in the case of the IP subsidy when comparing between district and provincial hospitals.

#### Benefit incidence by geographic region

**Figure 4:**
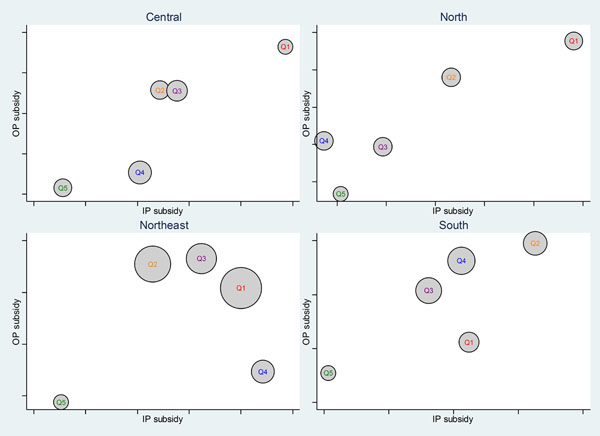
Government health subsidy for OP and IP services across wealth quintiles by geographic region, 2004. Note: Circle sizes are proportional to number of UCS beneficiaries for each wealth quintile in the region

The public subsidy for OP and IP services that benefited disproportionately more the poor UCS beneficiaries at the national scale held true even at the sub-national level. Figure 4 reiterates this finding. It should be noted that the poorest northeast region (left lower panel), has the largest size of poorest (Q1) and poor (Q2) UCS members compared to other regions. Consistently across four regions, the richest quintiles located at the position of having the lowest level of OP and IP subsidies, whereas the poorest quintiles located at the position of having the highest subsidy level, except for the south region. This reflects homogeneity of the UCS program implementation and outcome throughout the country.

### Explaining the pro-poor health subsidies

#### Heath utilization

Variation in health subsidy by the government is driven by utilization and net government subsidy; the net subsidy is the difference between unit costs and out-of-pocket payment by households. As there is no bias in unit cost subsidies between rich and poor quintiles--namely equal treatment and equal costs for the same conditions, the variation is therefore attributed by utilization and out of pocket payment.

**Figure 5:**
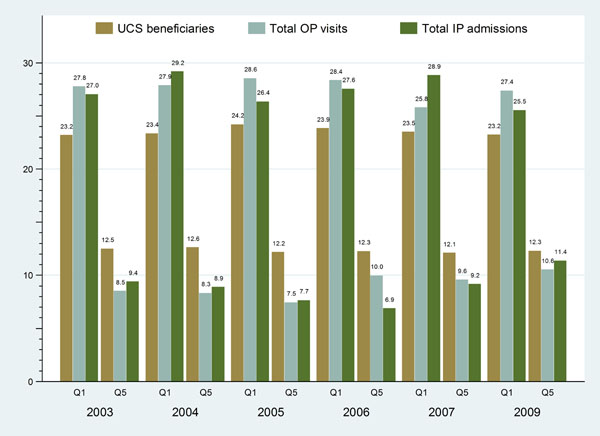
Distribution of OP visits and IP admissions by wealth quintile as compared with the UCS beneficiary distribution, 2003-2009

The subsidy distribution across the economic gradient is similar in both direction and degree to the utilization distribution in our analysis. The pro-poor government health subsidy is determined by pro-poor utilization where uses concentrated among the poor UCS members, see Figure 5.

Unsurprisingly, the poorest quintiles used more services than the richest quintiles compared to the size of beneficiaries in the poorest quintiles. The proportion of all-facility OP visits by the poorest quintile (26-29%) was higher than the corresponding population proportion (23-24% of total UCS members from 2003 to 2009. In contrast, the OP visits by the richest quintile (7-11%) were disproportionately lower than the size of its population, 12-13% of total UCS members.

Similarly, the poorest quintile UCS beneficiaries had a higher proportion of hospital admissions (26-29%) than the corresponding population size (23-24%) whereas the number of hospital admissions for the richest quintile where the size of population was 12-13% was disproportionately lower (7-11%).

When OP visits and admissions were concentrated among the poorest UCS beneficiaries more than their richest counterparts, this pro-poor utilization drove the pro-poor outcome of government health subsidies.

#### Health payment across rich-poor quintiles

During the UC period, certain UCS beneficiaries reported having to pay out-of-pocket for their medical services. As a result of the high frequency OP utilization, more UCS members, 4% to 9%, though small, paid for OP services than those for admission services, 1% to 4% when they chose to bypass services to higher level of care without properly referral; or paid for self-prescribed medicines in private pharmacies or used private providers not covered by the Scheme, Table 2. In public health facilities, 4-5% of the UCS members paid for their OP visits during 2003-2006, in contrast to the 1-2% who did so during 2007-2009 period when the 30 Baht flat rate copayment was abolished after the change of government.

**Table 2:** Probability of any health payment by type of health facility, %

	2003	2004	2005	2006	2007	2009
Any OP payment	7.2	8.9	8.4	7.2	5.2	4.4

Public facility	4.4	5.0	4.9	3.6	0.9	1.8

• Health center	1.8	2.2	2.0	1.2	0.3	0.8

• District hospital	1.7	1.9	2.0	1.6	0.3	0.5

• Provincial hospital	0.6	0.8	0.8	0.6	0.2	0.4

• University hospital	0.1	0.1	0.1	0.1	0.1	0.0

• Other govt. hospital	0.3	0.2	0.2	0.1	0.1	0.1

Private facility	3.0	4.1	3.7	3.8	4.3	2.7

• Private hospital	0.4	0.5	0.4	0.4	0.3	0.4

• Private clinic	2.6	3.6	3.3	3.5	4.0	2.3

Any IP payment	3.5	3.8	4.0	3.8	2.2	1.1

Public facility	2.9	3.2	3.5	3.4	1.8	0.8

• District hospital	1.7	1.7	1.9	1.8	0.9	0.3

• Provincial hospital	0.9	1.3	1.4	1.5	0.8	0.4

• University hospital	0.1	0.2	0.2	0.1	0.1	0.0

• Other govt. hospital	0.3	0.2	0.2	0.1	0.1	0.1

Private hospital	0.6	0.6	0.5	0.5	0.5	0.3

Note: Sampling weights applied

The payment for IP admission in public hospitals occurred for 3% of UC patients admitted during 2003-2006 and reduced to less than 2% in 2007 and 2009 when copay was abolished. Health payment to private facilities by the UCS members occurred mostly for OP visits to private clinics but rarely for private hospital visits and admissions, as they are more costly and unaffordable in particular private sector IP services.

**Figure 6:**
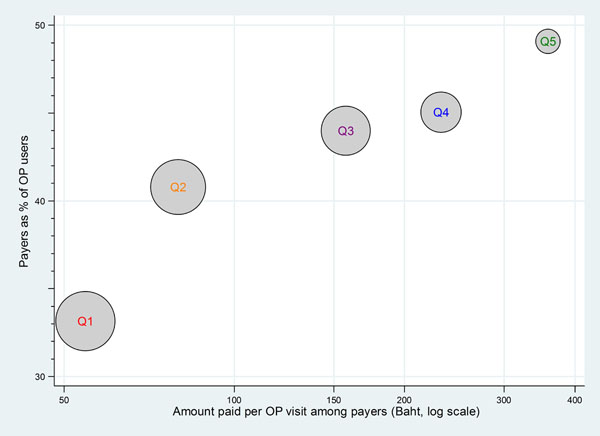
Payers and payment amount for OP public facility by wealth quintile, 2004. Note: Q1 –poorest 20% of population, Q5 –richest 20% of population. Circle sizes are proportional to number of the out-patients who were UCS beneficiaries for each quintile

**Figure 7:**
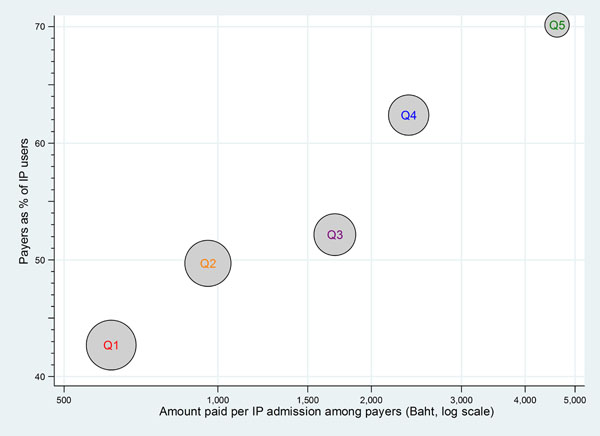
Payers and payment amount for IP public facility by wealth quintile, 2004. Note: Q1 –poorest 20% of population, Q5 –richest 20% of population. Circle sizes are proportional to number of the in-patients who were UCS beneficiaries for each quintile

The difference in likelihood and average amount of payment to public facilities across wealth quintiles of the UCS beneficiaries for OP and IP services is illustrated in Figures 6 and 7, respectively. The size of the balloon reflects the size of the population in the five wealth quintiles. A common pattern emerged for all years; Figure 6 and 7 illustrated 2004.

Figure 6 shows out of pocket payment for OP services, where 33% of the poorest quintiles paid for OP services but the amount was small, slightly more than 50 Baht average per visit (less than US$ 2) while around 50% of the richest quintiles paid for their OP services with a much larger average amount, almost 400 Baht per visit (approximately US$ 13).

Similarly, Figure 7 illustrates that a lower proportion of the poorest compared to the richest quintiles were paying for IP services with a much lower average amount per admission of 600 Baht (approximately US$ 20) compared to almost 5,000 Baht (approximately US$ 167) respectively.

## Discussion

This study provides clear and consistent evidence of pro-poor government health budget subsidies for OP and IP services between 2003 and 2009, which preferentially benefited the poorer quintiles compared to the relative size of their respective population. Three contributing factors explain the pro-poor government health subsidies.

First, the pro-poor utilization is the result of improved access to heath services provided by district health systems for the poor, a majority of who reside in rural areas. The district health system consists of a 30 to 90 bed district hospital and an average of 10 health centres per approximately 50,000 populations in the catchment area. Health centres and district hospital form a contractor provider network for UCS members through contractual arrangement with the National Health Security Office. This network provides a comprehensive set of OP and IP services to UCS members. The fully functioning district health system is a strategic hub of achieving pro-poor utilization for OP and IP due to its geographical proximity, so called “close-to-client services” where indirect cost of travelling and access is low [9]. District health systems are functioning due to adequate budget support, three years mandatory rural health services by all new graduate doctors since 1972, and later extended to cover nurses, dentists, pharmacists and other paramedics [10]. Such rural mandatory policies have resulted in a substantial reduction in regional gaps in the density of human resources over the last four decades [11]. Also the availability of private health providers for the rich reduces competition from the rich for services provided by public sector.

Second, the very low level of out-of-pocket payment is the result of two factors; a comprehensive benefit package entitled to all UCS members including OP, IP which covers all medicines with reference to the national drug list, high cost care such as chemotherapy, radiation therapy, prevention and health promotion services. Although there was a minimum level of flat rate copayment of 30 Baht (US$ 1) per visit or per admission; this was terminated in 2006. The out-of- pocket payments are for services either not covered by the benefit package such as private clinics or bypassing the registered providers without referral procedures. The minimum level of out-of-pocket payment are in favour of the poor; this is reflected by the probability and level of payment for both OP and IP services is consistently low among the poorer than the richer quintiles.

Our additional analysis found the 88-96% of UCS members actually used UC services; the poorest quintile had higher rate of using their entitlements, 70-80% for OP visit and 90-95% for admission, while the among the richest counterparts, 40-60% of them used OP entitlement, 45-80% used IP entitlement [12].

Third, government financial commitment was significant, not only the rhetoric in Parliament or at press conferences. The 36.2% real term increase between 2003 and 2009 is significant. Evidence from National Health Account shows the general government expenditure on health increased from 50% to 67% of total health expenditure, and out of pocket payment reduced from 33% in 2001 to 18% of in 2008. The low level of out of pocket payment is on par with the average of OECD countries. Despite a favourable benefit package, share of total health expenditure in GDP is minimum, increased from 3.3% of GDP in 2001 to 4.0% in 2008. The per capita health spending was US$ 61 in 2001 and US$ 173 in 2008 [13].

It is interesting to see if the pro-poor public subsidy and health utilization have been translated into an equitable achievement of health outcomes. National IP dataset shows that the top-80% deadly diseases and the conditions of which death is amenable to health care revealed no increasing trends in both in-hospital and 30-day mortalities over the post-UCS period [14]. Our additional analysis of the same HWS datasets found 19-26% of the poorest quintiles of the UCS members reported at least one illness episode during a prior month, whereas 14-19% of the richest quintile did so; reflecting higher health needs among the poor. Even after controlling for a higher health need among the poorer population, health services provided by district health systems still disproportionately concentrated among the poor [15].

The pro-poor government health spending is homogeneously distributed across four geographical regions; this is a result of the homogeneity of district health systems development nationwide. The rural mandatory services are enforced to the whole country with financial incentives such as hardship allowance, lump sum per diem, non-private practice incentives and workload allowance as well as other non-financial incentives such as housing and social recognition. These interventions are effective and recommended by WHO for rural retention [16].

Countries with high level of out of pocket payment and no effective policies protecting the poor from health payment have benefit incidence is in favour of the rich. For example the poorer groups in Vietnam [17] get much less than their population share of hospital-based care and other public care but more than a proportionate share of care provided at commune health centers. Pro-rich bias in the distribution of hospital-based care is a common finding across several countries [18-21]. Government health spending in African countries was in favour of the rich; for example, the poorest quintiles in Ghana benefited 10%, 13% and 11% at primary facilities, hospital outpatient and inpatient services respectively; while the richest Ghanaian’s benefited 31%, 35% and 32% at these facilities. Also similar findings were reported from Kenya, Tanzania, Madagascar and Guinea [22]. The Indonesian poorest quintiles benefited 7% and 5% from hospital outpatient and inpatient services while the richest counterparts all benefited 41% from these services.

Among eleven countries in Asia, with the exception of Hong Kong, Malaysia, Sri Lanka and Thailand, the poor get much less than their population share of the public health subsidy. The pro-poor benefit incidence in some of these Asian countries are the results of limiting the use of user fees, effective protection of the poor from payment, and building a wide network of health facilities [23] so that the poor can effectively use these services.

## Conclusion

This paper clearly illustrates that a good design of UCS, the public health insurance scheme, results in equity outcome, in favour of the poor as measured by benefit incidence analysis. Purchasing services at “close-to-client” provider, in this case the primary healthcare network at district level is a good practice, where rural poor can actually use services when needed and with better utilization rate. A comprehensive benefit package and minimum or zero copayment results in low level of out of pocket payment.

## Competing interest

The authors declare they have no competing interests.

## Authors’ contributions

All authors involved in conceptualization of the article, SL analyzed and interpret the results. SL and VT produced the first draft. All authors commented, revised, finalized and approved the manuscript.
